# Precision EDM of Micron-Scale Diameter Hole Array Using in-Process Wire Electro-Discharge Grinding High-Aspect-Ratio Microelectrodes

**DOI:** 10.3390/mi12010017

**Published:** 2020-12-26

**Authors:** Zhixiang Zou, Zhongning Guo, Qinming Huang, Taiman Yue, Jiangwen Liu, Xiaolei Chen

**Affiliations:** 1 State Key Laboratory of Precision Electronic Manufacturing Technology and Equipment, Guangdong University of Technology, Guangzhou 510006, China; zouzx606@163.com (Z.Z.); znguogdut@163.com (Z.G.); huangqinming123@163.com (Q.H.); 2 Guangzhou Key Laboratory of Nontraditional Machining and Equipment, Guangdong University of Technology, Guangzhou 510006, China; 3 The Advanced Manufacturing Technology Research Centre, Department of Industrial and Systems Engineering, The Hong Kong Polytechnic University, Hung Hom, Hong Kong, China; tm.yue@connect.polyu.hk

**Keywords:** micro-EDM, high-aspect-ratio microelectrode, microelectrode wear, micro-hole array

## Abstract

Micro-electrical discharge machining (micro-EDM) is a good candidate for processing micro-hole arrays, which are critical features of micro-electro-mechanical systems (MEMS), diesel injector nozzles, inkjet printheads and turbine blades, etc. In this study, the wire vibration of the wire electro-discharge grinding (WEDG) system has been analyzed theoretically, and, accordingly, an improved WEDG method was developed to fabricate micron-scale diameter and high-aspect-ratio microelectrodes for the in-process micro-EDM of hole array with hole diameter smaller than 20 μm. The improved method has a new feature of a positioning device to address the wire vibration problem, and thus to enhance microelectrodes fabrication precision. Using this method, 14 μm diameter microelectrodes with less than 0.4 μm deviation and an aspect ratio of 142, which is the largest aspect ratio ever reported in the literature, were successfully fabricated. These microelectrodes were then used to in-process micro-EDM of hole array in stainless steel. The effects of applied voltage, current and pulse frequency on hole dimensional accuracy and microelectrode wear were investigated. The optimal processing parameters were selected using response–surface experiments. To improve machining accuracy, an in-process touch-measurement compensation strategy was applied to reduce the cumulative compensation error of the micro-EDM process. Using such a system, micro-hole array (2 × 80) with average entrance diameter 18.91 μm and average exit diameter 17.65 μm were produced in 50 μm thickness stainless steel sheets, and standard deviations of hole entrance and exit sides of 0.44 and 0.38 μm, respectively, were achieved.

## 1. Introduction

Micro holes are widely applied in the fields of micro-electro-mechanical systems (MEMS) [1], inkjet printheads [2], diesel injector nozzles [3] and aeronautical engines [4], etc. For example, structures with micro holes have been used to aid cooling in aeronautical engine components such as turbine blades. Presently, various micro-machining methods, such as micro mechanical drilling, laser drilling, electrochemical drilling (ECD) and micro-electrical discharge machining (micro-EDM) have been adopted to machine micro-holes [5,6,7,8]. However, each method has its limitations in cost, machining efficiency, properties of the workpiece and aspect ratio of a micro-hole [9]. For example, Routio and Saynatjoki [10] observed that the micro mechanical drill performance is affected by drill material, drill point geometry and the workpiece materials. Nasrollahi et al. [11] pointed out that there are some limitations, such as achievable aspect ratio, tapered walls and material spatters in laser micro drilling. In addition, the debris and burrs are also inevitable due to the ablation and melt expulsion coexist in laser machining process [12]. As for the ECD of micro-hole array, under multiple holes ECD conditions, uneven electrolyte volume divided in each tube electrode would probably affect the drilling stability and the uniformity of hole size, as reported by Fang et al. [13]. Among these micro-machining methods, micro-EDM perhaps is the most promising process for creating micro-hole arrays. The main advantage of this method is that it can engage ultra-small-diameter electrodes to produce extremely small holes with diameter of several tens of microns or even smaller. This is mainly due to the non-contact machining nature of the EDM method, and as such the force between the electrode and workpiece is negligible [14]. 

In micro-EDM, there are two common ways to create micro-hole array, using arrayed microelectrodes or a single microelectrode. Using the LIGA technology, Takahata and Gianchandani [15] have fabricated array of 400 Cu micro-electrodes. The electrode diameter and length were 20 and 300 μm, respectively, and employing EDM using these electrodes, micro through-hole array in 50 μm thick stainless-steel sheets were successfully produced. On the other hand, Chen et al. [16] has fabricated an array of 10 × 10 micro squared electrodes with high aspect ratio using the micro-EDM method. The width and height of the electrode were 21 and 700 μm, respectively, and they managed to use the electrode array to produce an array of 900 micro through-holes in 30 μm thick stainless-steel sheets. Hu et al. [17] combined UV-LIGA and micro-EDM to fabricate microelectrode array with the electrode diameter of 100 μm and a high aspect ratio of 10. Taking a different approach, Kim et al. [18] employed the reverse EDM method to fabricate various multiple microelectrodes, and micro-hole array of various shapes were machined on stainless steel. Though using an electrode array achieves a high machining efficiency, the problems of machining stability and poor dimensional accuracy of the machined micro-holes still cannot be easily overcome. This is primarily due to the fact that the array electrode cannot be rotated, and thus, the EDM debris cannot be readily discharged out of the hole during machining. As a result, an unstable machining state is often encountered. Meanwhile, each electrode of the array inevitably introduces machining error because each individual electrode has a different degree of tool wear, and this will cause dimensional inaccuracy of the machined array. Moreover, the fabrication of array electrodes is a very time-consuming operation.

In parallel to the advances made in the research of using array electrodes, considerable research results have shown that by utilizing a single electrode to produce micro-hole array, not only the electrode fabrication step is much simpler, a more stable EDM processing condition is obtained with the rotation of the electrode. Although rapid electrode wear is still unavoidable in EDM, an electrode with a high aspect ratio (i.e., a long electrode) can be used to produce many holes before changing of a new electrode is required. Indeed, much effort has been devoted to developing various methods to fabricate high-aspect-ratio microelectrodes. Jahan et al. [19] fabricated the microelectrode with 40 µm in diameter and 75 in aspect ratio by block electrode discharge grinding (BEDG) method. Gil et al. [20] presented a new configuration of the block EDG electrode process, named inverse slab electrical discharge milling (ISEDM), by using this method, the microelectrode with 200 μm in diameter and 90 in aspect ratio was obtained. However, due to the explosive force, which will cause taper error in the axial direction during BEDG process, this method is not suitable for fabricating the microelectrode with diameter less than 100 μm. Yin et al. [21] proposed electrical discharge machining grinding using two block electrodes (EDG-TBE) to relieve the electrical discharge explosive force influence on the microelectrode fabrication accuracy, and the microelectrode with 46 μm in diameter and 27 in aspect ratio was fabricated with this method. Among the microelectrode fabrication methods, the wire electro-discharge grinding (WEDG), which was invented by Masuzawa et al. [22], is one of the most promising processes for fabricating high-aspect-ratio microelectrodes. By employing this method, Li et al. [23] successfully fabricated microelectrodes with diameter of 25 μm and aspect ratio of 20. To improve dimensional accuracy, Zhang et al. [24] proposed a tangential feed WEDG (TF-WEDG) method to avoid the grinding positioning error, the microelectrode with 64 μm in diameter and 12.5 in aspect ratio was fabricated, and the repeated machining accuracy of microelectrodes was less than 2 μm; whilst Li et al. [25] adopted a novel active supplying wire-electro discharge grinding (AS-WEDG) device to reduce the wire fluctuation at the grinding location, and the microelectrode with 40.3 μm in average diameter and 49.6 in aspect ratio was successfully fabricated.

Notwithstanding the advancements in the fabrication of micron-sized hole array, only a few published papers relating to using a single electrode with high-aspect-ratio to machine micro-hole array with hole diameter smaller than 20μm (which are in great demand in printer nozzles) can be found in the literature. A recent article discussed the use of a single electrode to produce micro-hole array; however, the aspect ratio of the electrode was not high (only 49) and the hole diameter was relatively large (about 45 μm), as described by Li et al. [25]. In view of the limitations of the current technologies, this study aims to develop a method and a fabrication strategy for producing high-aspect-ratio microelectrodes for micro-EDM of micro-hole array with hole diameters of less than 20 μm.

## 2. The Improved WEDG Method and Microelectrodes Fabrication Process

### 2.1. The Improved WEDG Method

In order to achieve a high processing efficiency in micro-EDM of micro-hole array using a single microelectrode, a relative long (high-aspect-ratio) microelectrode is necessary. The WEDG is one of the most useful methods for fabricating microelectrodes. Notwithstanding the merits of the WEDG method, there are still problems needed to be solved and improvements to be made before it can be effectively utilized for fabricating micro-electrodes with a high aspect ratio. The main problem encountered is wire electrode vibrations, which involved vibrations of the wire transport system and the grinding location [25]. This vibration problem severely affects the dimension accuracy of the micro-electrode. It is envisaged that in EDM of small holes, say (<20 μm), a high electrode wear rate would be encountered. To avoid the need of frequently changing the electrode so as to guarantee the machining efficiency in micro-EDM, a high-aspect-ratio electrode is required, but unfortunately to fabricating high-aspect-ratio microelectrodes with this diameter (<15 μm) is extremely difficult due to the vibration problem. Hence, the vibration issues of the wire electrode transport system should be addressed.

**Figure 1:**
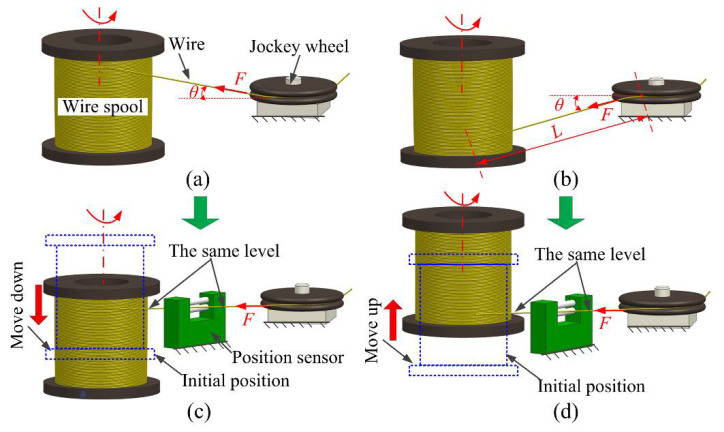
Schematic diagrams illustrate the angle between the wire and the jockey wheel: (**a**,**b**) without the position sensor ($\theta \neq 0^{0}$), (**c**,**d**) with the position sensor ($\theta =0^{0}$).

During WEDG process, it is assumed that the wire produces vibration under the action of the excitation source, the wire electrode length and density are L and ρ, respectively. The pull force of the wire spool is F and the angle between the pull force and the horizontal direction is θ (see Figure 1a,b). The wave velocity through the wire can be described by Equation (1) [26]:

(1) $$a=\sqrt{\frac{F\cos\theta }{\rho }}$$

In this study, it is assumed that the excited vibration of the wire is u(L,t)=Asinωt, where A is a constant, ω is the excitation frequency, and t is the time. Assuming the influences of other forces are negligible. Therefore, the corresponding definite solution problem can be expressed by Equation (2):

(2) $$\{\begin{array}{ccc} \frac{\partial^{2}u}{\partial x^{2}}-\frac{1}{a^{2}}\frac{\partial^{2}u}{\partial t^{2}}=0 & 0<x<L & t>0\\ u{|}_{x=0}=0^{} & u{|}_{x=L}=A\sin\omega t & t>0\\ u{|}_{t=0}=0^{} & \frac{\partial u}{\partial t}{|}_{x=L}=0 & 0\leq x\leq L \end{array}$$

The vibration equation of the wire electrode, i.e., Equation (3) can be obtained by using the variables separation method to solve Equation (2):

(3) u(x,t)=Asin(ωxa)sin(ωLa)sinωt+∑n=1∞2AaωL(−1)n(ωn2−ω2)L2sinnπatLsinnπxL

where $\omega_{n}$ is the natural frequency of the wire electrode. Equation (3) shows that the wire electrode vibration is related to both the wire electrode’s natural frequency $\omega_{n}$ and the excitation frequency ω. The wire electrode vibration decreases with the wire increasing of the electrode’s natural frequency $\omega_{n}$, which can be described using Equation (4):

(4) $$\omega_{n}=\frac{n\pi a}{L}=\frac{n\pi }{L}\sqrt{\frac{F\cos\theta }{\rho }}\;\left(n=1,2,3\cdots \right)$$

Clearly, the wire electrode’s natural frequency $\omega_{n}$ achieves its maximum value when θ = 0°, i.e., the wire pay-off positions of the wire spool and the jockey wheel are at the same horizontal line. This condition produces the smallest wire electrode vibration (Equation (3)). With this in mind, an improved WEDG system for improving microelectrode dimensional accuracy and enhancing aspect ratio was developed. It consists of a positioning sensor. The major function of this sensor is to keep the wire pay-off positions of the wire spool and the jockey wheel at the same horizontal position during the entire WEDG process (see Figure 1c,d). 

**Figure 2:**
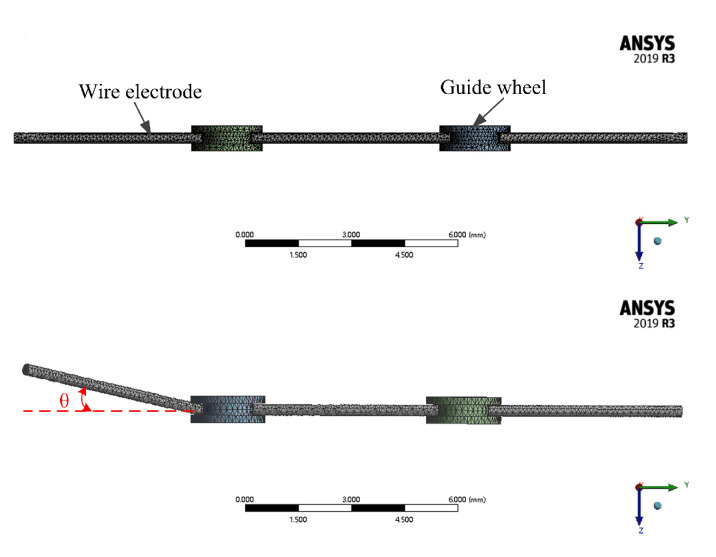
Simplified simulation model.

**Figure 3:**
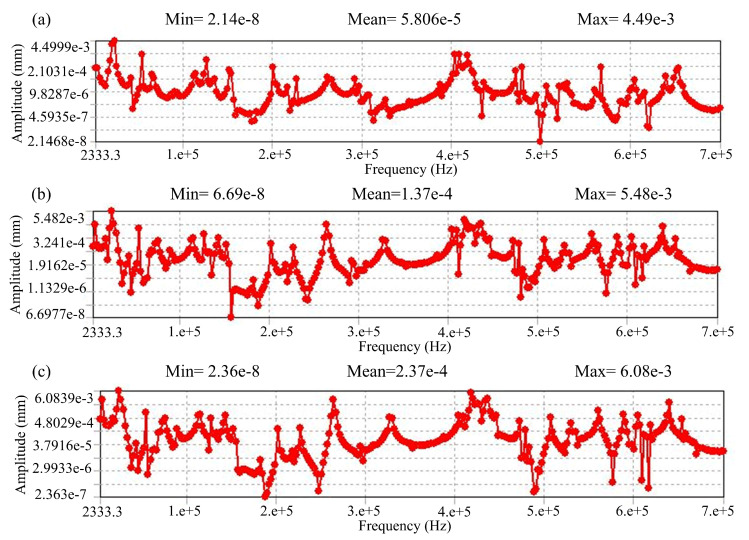
The wire electrode vibration amplitude with different theta: (**a**) θ is 0°, (**b**) θ range from 0° to 15°, and (**c**) θ range from 0° to 30°.

To justify the validity of the model, the vibration of the working wire electrode between the fixed guider has been simulated by ANSYS (ANSYS software2019, Pittsburgh, PA, USA) (see Figure 2). The pull force is assumed to be 35 N, the brass wire electrode diameter is 200 μm, and the measured wire length between the guider is 7 mm. The simulation results clearly show that the smallest mean vibration amplitude of the wire electrode can be obtained when the θ is 0° (see Figure 3).

**Figure 4:**
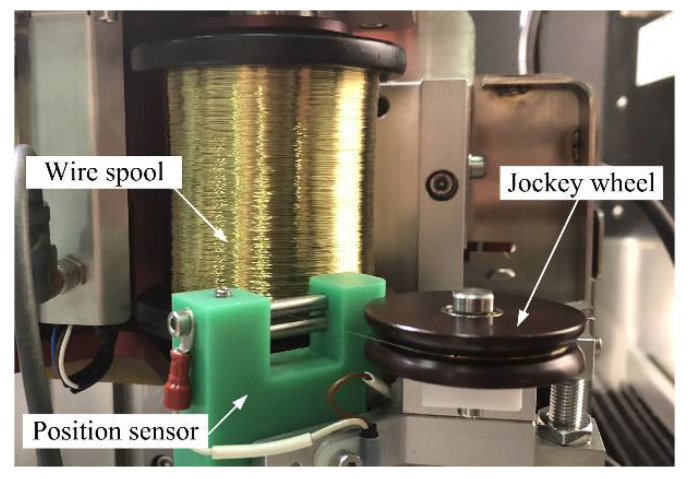
Photo of the improved wire electro-discharge grinding (WEDG) device.

When in operation, once the wire touches the positioning sensor ($\theta \neq 0^{0}$), a trigger signal will be sent to the spool movement controller. As a response, the spool movement controller will regulate the spool until with its wire pay-off positions adjusted to the same horizontal position as the jockey wheel, i.e., $\theta =0^{0}$ (see Figure 1c,d). As a result, wire electrode vibrations caused by the horizontal position difference between the wire pay-off positions of the wire spool and the jockey wheel would be weakened. A photo of the improved WEDG device is shown in Figure 4.

### 2.2. Microelectrodes Fabrication

**Figure 5:**
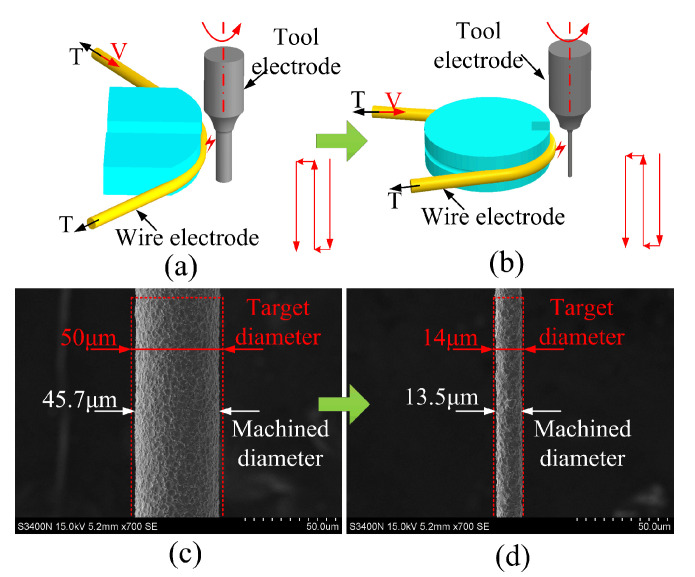
Schematic diagrams showing the two-step WEDG fabrication process: (**a**) initial grinding, (**b**) precision grinding, (**c**) SEM image of the initial grinded microelectrode, (**d**) SEM image of the precision grinded microelectrode.

A 400 μm diameter tungsten carbide (WC) rod was used as the initial tool electrode and the brass wire electrode for WEDG of the tool electrode has a diameter of 200 μm. In WEDG, the tungsten carbide electrode was of positive polarity, while the wire electrode was of negative polarity. The spindle speed is at 600 rpm, the wire speed is at 9mm/s, the wire tension is 35 N, and hydrocarbon oil was used as the working fluid. To improve the fabrication efficiency and quality of the microelectrode, a two-step fabrication process was adopted (Figure 5a,b). The fabricated microelectrode diameters were measured using laser confocal microscopy (OLS4000; Olympus, Tokyo, Japan).

In Step 1, i.e., initial grinding, rough machining of the WC rod to the target diameter (50 ± 5 μm) with high machining efficiency was obtained using a high applied voltage of 130 V, meaning a high discharge energy condition (pulse frequency 120 kHz, pulse width 5 μs). An SEM image of an initial grinded electrode is shown in Figure 5c. For Step 2, a smaller discharge energy condition was used (applied voltage 80 V, pulse frequency 160 kHz and pulse width 1 μs). After Step 2, the microelectrode was finished to the target diameter of 14 ± 1 μm (see Figure 5d).

**Figure 6:**
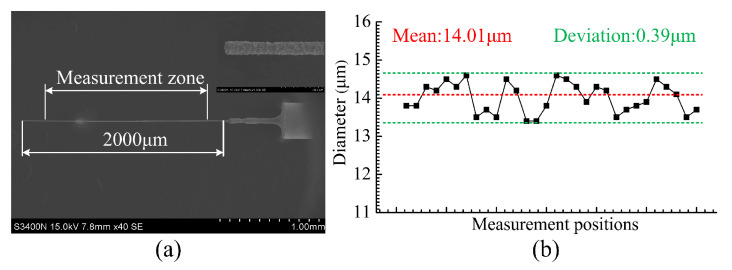
(**a**) SEM image of the microelectrode produced using the improved WEDG method, (**b**) measurements of the electrode diameter.

Figure 6a shows the SEM image of the whole microelectrode fabricated using this improved WEDG method. The 2 mm length microelectrode has an average diameter of 14.01 μm, i.e., a high aspect ratio of 142 was achieved. Along the whole length of the microelectrode, the diameter deviation was only 0.39 μm (results shown in Figure 6b). These results indicate that electrodes with ultra-small diameter (<15 μm), high aspect ratio (>140) and small deviation in diameter can be successfully fabricated using our improved WEDG method.

## 3. The Influence of Pulse Parameters on the Micro-Hole Diameter and Electrode Wear

Prior to micro-hole array machining, the effects of voltage, current and pulse frequency on geometric accuracy and quality of the single hole and electrode wear were firstly studied. The experimental tests were conducted using a SARIX-200hpm micro-EDM machine. The workpiece was 50 μm thick stainless steel. The microelectrode (14.01 μm average diameter and 142 aspect ratio) which was previously fabricated using our improved WEDG method (Section 2.2) was used as tool electrode. The micro-hole diameters were measured using laser confocal microscopy (OLS4000; Olympus, Tokyo, Japan). The micro-hole surface morphology was analyzed via scanning electron microscope (S-3400N; Hitachi, Tokyo, Japan). The wear length of the electrode is measured by the machine touch measurement method. Each set of the experiment was repeated five times; the other experiment conditions are given in Table 1.

### 3.1. Effect of the Applied Voltage and Current on Hole Diameter and Electrode Wear

**Figure 7:**
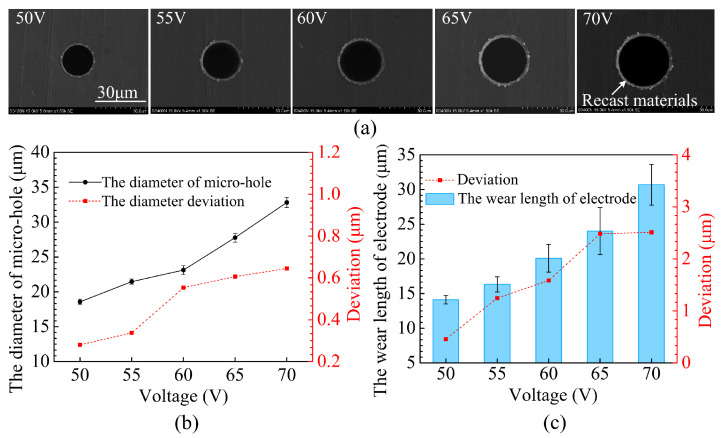
The effect of the pulse voltage on (**a**) micro-hole entrance, (**b**) micro-hole diameter, (**c**) electrode wear.

Micro-holes were fabricated with different pulse voltages (V) of 50, 55, 60, 65 and 70, whilst frequency, pulse width and current were fixed at 250 kHz, 3 μs and 80, respectively. Figure 7a shows the SEM images of the entrance of the holes. Using a higher voltage would generate larger electrical spark energy, hence more workpiece material would be melted, this has led to more recast material being deposited around the rim of the hole (see Figure 7a). The recast materials could affect the mechanical properties of the machined part. The effects of pulse voltage on micro-hole diameter and electrode wear can be realized from Figure 7b,c. When pulse voltage was increased from 50 to 70 V, the mean diameter of the micro-hole increased from 18.57 to 32.82 μm, and the diameter deviation increased from 0.28 to 0.65 μm. Whereas, the average wear length of the tool electrode increased from 14.11 to 30.69 μm, and the electrode wear deviation was also increased. The results indicate that under a high pulse voltage condition, the dimensional accuracy of the micro-hole has fallen. In fact, electro-thermal melting and evaporation are the main materials removal mechanisms of micro-EDM process. Apparently, a higher voltage means more intense electro-thermal melting and evaporation activities, and hence more workpiece materials will be removed. As a result, a larger micro-hole and more severe electrode wear will be produced. Electrode wear would cause diameter fluctuation of the micro-hole. On the other hand, the non-contact forces acting at the surface of the tool electrode, such as the electrostatic force, the electromagnetic force and the pressure of bubbles generated by evaporation and dissociation of the dielectric liquid also increase with an increase of voltage. A higher acting force on the micro-electrode will intensify oscillation of the electrode and may even resulted in distortion of the electrode, and this would lead to large diameter deviation.

**Figure 8:**
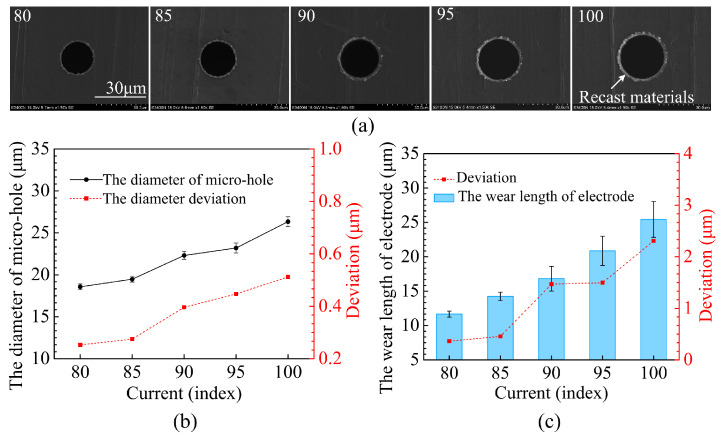
The effect of the current on (**a**) micro-hole entrance, (**b**) micro-hole diameter, (**c**) electrode wear.

A similar effect of voltage on hole diameter and dimensional deviation was also noted with current, since a higher voltage or current produces a higher energy input. Figure 8a shows the top surfaces of the micro-holes, while Figure 8b,c show the effect of current on the micro-hole diameter and degree of electrode wear for processing currents of 80, 85, 90, 95 and 100. The frequency, pulse width, and voltage were fixed at 250 kHz, 3 μs, and 50 V, respectively. When the current was increased, the micro-hole diameter became larger and the machined surface quality worsened. As the current increased from 80 to 100, the mean micro-hole diameter increased from 18.57 to 26.34 μm and the deviation in diameter increased from 0.25 to 0.51 μm. The average wear length of the tool electrode increased from 13.71 to 25.44 μm and the electrode wear deviation increased from 0.36 to 2.31 μm.

### 3.2. Effect of the Pulse Frequency on Hole Diameter and Electrode Wear

**Figure 9:**
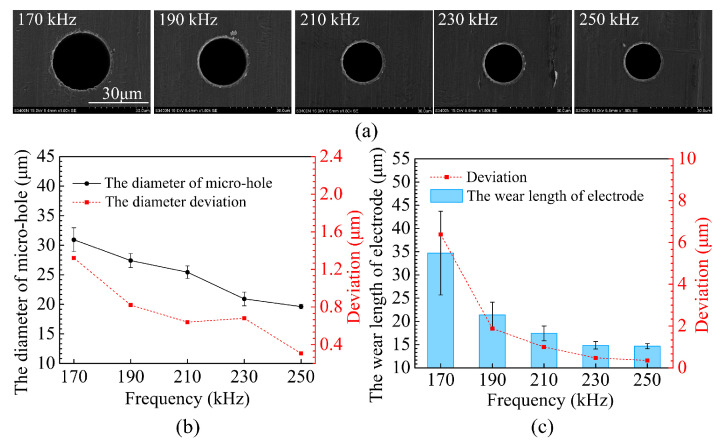
The effect of the pulse frequency on (**a**) micro-hole entrance, (**b**) micro-hole diameter, (**c**) electrode wear.

Figure 9 reveals the effects of the pulse frequency on hole diameter and electrode wear with a current of 80, a pulse width of 3 μs and a voltage of 50 V. One would expect that, decreasing the pulse frequency would decrease the hole diameter and reduce electrode wear, as a smaller energy input is provided. Similar effects are noted with voltage and current. However, both the hole diameter and electrode wear increase slightly when the pulse frequency decreases from 250 kHz to 210 kHz. Both quantities then increase greatly when the pulse frequency decreases from 210 kHz to 170 kHz. Although the reasons for this remain unclear, it is considered that resonance may play an influential role. During micro-EDM, with a pulse power supply, the forces (e.g., the electrostatic and electromagnetic forces) generated by the discharge vibrate the microelectrode. When the vibration excitation frequency approaches the electrode resonant frequency, strong electrode vibration occurs. In this study, electrode vibration may intensify when the pulse frequency decreases from 210 to 170 kHz. The increased electrode vibration would both enlarge the hole dramatically and cause rapid electrode wear. Thus, it appears that one must avoid electrode resonance in order to benefit from the high accuracy and low electrode wear of the micro-EDM.

### 3.3. Response Surface Experiment on Hole Diameter and Electrode Wear

**Figure 10:**
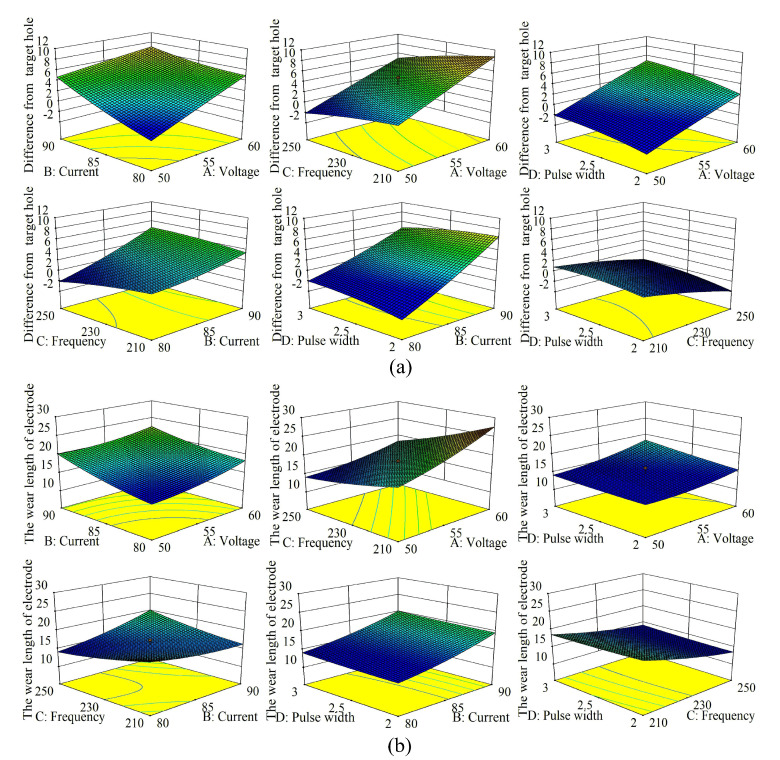
The influences of pulse parameters on (**a**) micro-hole diameter, (**b**) electrode wear.

Based on the results of the single-factor experiments discussed above, specific pulse parameter values were selected for response–surface experiments. Analyzing the interactions among the different pulse parameters can lead to an optimum machining condition. A previously produced 2 mm long microelectrode with an average diameter of 14.01 μm is used for EDM machining of micro-hole array in this experiment (Section 2.2) and the target micro-hole diameter was 19 μm. Figure 10 shows interactions between the voltage, current, pulse frequency, pulse width and micro-hole diameter (see Figure 10a) and electrode wear (see Figure 10b).

The results show that the condition of applied voltage of 50 V, current of 80, pulse frequency of 250 kHz and pulse width of 3 μs is considered to be optimal when machining accuracy and electrode wear are the decisive factors.

## 4. Microelectrode Wear Compensation

**Figure 11:**
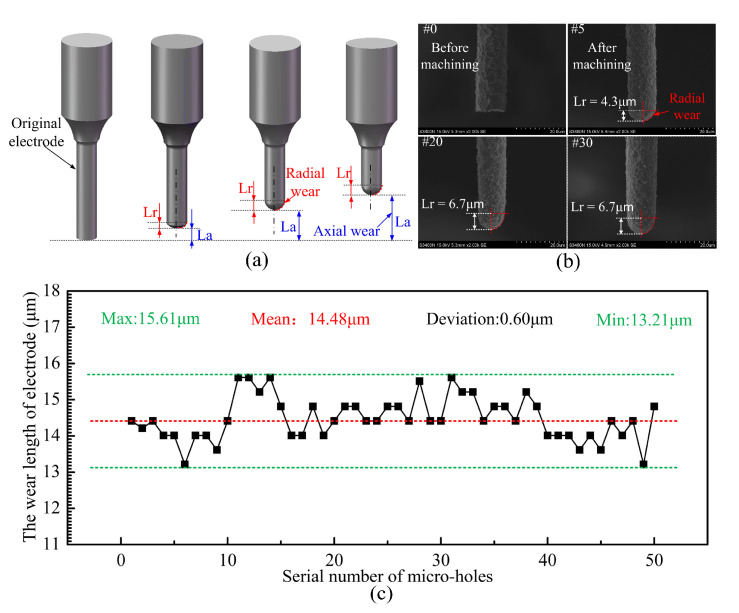
(**a**) Schematic diagrams showing the evolution process of microelectrode wear, (**b**) the microelectrode tip geometry changes after machining a certain number of micro-holes, and (**c**) microelectrode wear length variation during micro-hole array machining process.

During the micro-EDM process, electrode wear is inevitable. Wear occurs in both the axial ($L_{a}$) and radial directions ($L_{r}$) (Figure 11a), this not only severely affects dimensional accuracy but also reduces the consistency accuracy of micro-hole array. Obviously, wear compensation of the microelectrode also should be investigated prior to micro-hole array machining.

Figure 11b shows the shapes of the microelectrodes after drilling a series of holes. The sharp edge of the microelectrode tip gradually changed to a pointed parabolic shape after drilling of 20 holes (#20). Therefore, it is important to consider the wear of the microelectrode in the radial direction ($L_{r}$). Figure 11c shows axial wear of the electrode after each hole is processed (the processing parameters are, the pulse voltage: 50 V, current: 80, pulse frequency: 250 kHz, the pulse width: 3 μs). The mean wear length of the microelectrode was about 14 μm, which was ten times higher than that of the electrode with large diameters (>40 μm). In addition, the microelectrode wear length has a large fluctuation.

**Figure 12:**
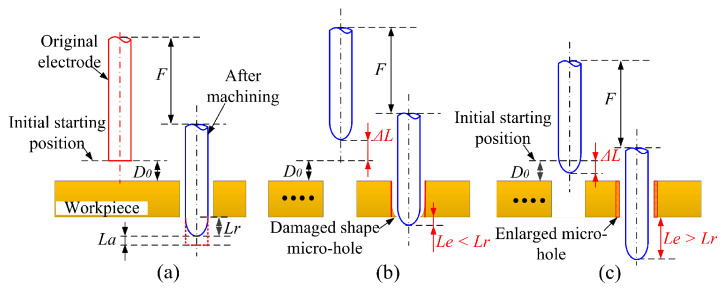
Schematic diagrams showing the effects of microelectrode wear on hole geometries: (**a**) initial condition, (**b**) hole geometry when ΔL>0, (**c**) hole geometry when ΔL<0.

The schematic diagram of Figure 12 shows the effect of microelectrode wear on hole geometries. In order to assure machining accuracy, the axial direction wear ($L_{a}$) and radial direction wear ($L_{r}$) could be compensated by the axial feed (F) (see Figure 10a), and the required axial feed distance can be calculated by Equation (5):

(5) $$F=D_{0}+S+L_{r}+L_{c}$$

where $D_{0}$ is the distance between the initial starting point and the workpiece surface, S is the workpiece thickness, $L_{r}$ is radial direction wear and $L_{c}$ is the fixed compensation length. During machining of the micro-hole array, the microelectrode wear length has a large fluctuation (see Figure 11c). Using the traditional fixed-length compensation method, the compensation error (ΔL) will be cumulated during machining process and can be determined by Li et al. [25]:

(6) $$\Delta L=\sum_{i=1}^{n}L_{a}^{i}-nL_{c}$$

where $L^{i}{}_{a}$ is axial wear length of the $i^{th}$ mcro-hole, n is the number of machined micro-holes. When ΔL>0, that is to say, $L_{a}$ is greater than $L_{c}$, the next machined hole shape accuracy will be destroyed by the electrode parabolic shape as illustrated in Figure 10b. On the contrary, if ΔL<0, the actual penetration distance ($L_{e}$) will be larger than the radial direction wear ($L_{r}$) (see Figure 12c). As a result, the micro-hole would be enlarged due to the extended machining time and the electrode vibration.

**Figure 13:**
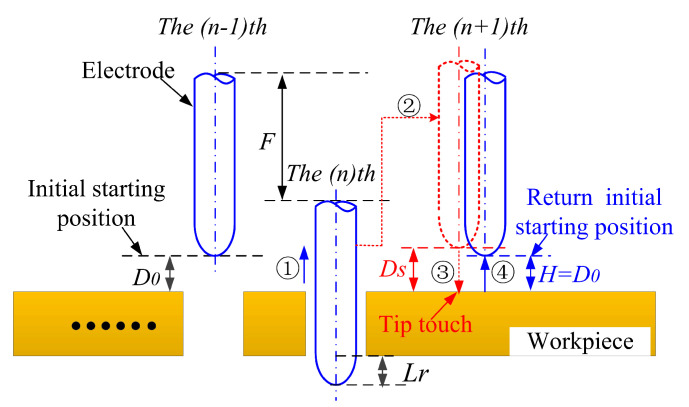
A schematic diagram illustrating the touch measurement compensation strategy.

**Figure 14:**
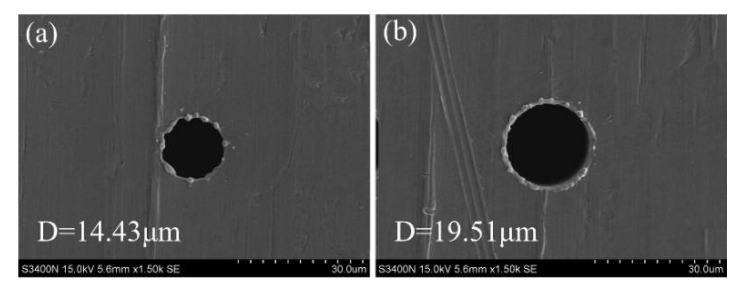
The image of the micro hole: (**a**) without compensation process, and (**b**) with the compensation process.

In order to solve this problem, this study proposes a touch measurement compensation strategy to eliminate the cumulative compensation error. As illustrated in Figure 13, an initial starting point is set before the start of processing and the distance between the initial point and the workpiece surface ($D_{0}$) is recorded. After machining each micro-hole, the microelectrode will be lifted up to a safe distance ($D_{s}$), and then allocated to the next hole position. Once it reaches the set position, the microelectrode moves toward the surface of workpiece. When the microelectrode tip touches the workpiece surface, a trigger signal will be sent to the spindle controller and the real-time coordinates will be record. Then, the spindle will move the microelectrode up until the distance between the workpiece and the tip point of the microelectrode reach $D_{0}$ (i.e., ΔL=0) (see Figure 13). Obviously, by utilizing this touch measurement compensation strategy, the cumulative compensation error (ΔL) associated with the traditional compensation method will be eliminated. The figure of the micro hole before and after the compensation process were shown in the Figure 14.

## 5. Micro-Hole Array Machining

A 50 μm thickness stainless steel sheet was used as the workpiece. The microelectrode (14.01 μm average diameter and 142 aspect ratio) which was previously produced using our improved WEDG method (Section 2.2) was selected as the microelectrode for EDM machining of micro-hole array. The processing conditions for micro-EDM were radial direction wear ($L_{r}=6.7_{}\mu m$), fixed compensation length ($L_{c}=16_{}\mu m$) and an initial starting distance ($D_{0}=20_{}\mu m$). A touch measurement compensation strategy was adopted to eliminate the cumulative compensation errors. The optimal machining parameters obtained from response–surface experiments (Section 3.3) are used for machining of micro-hole array, which are given in Table 2.

**Figure 15:**
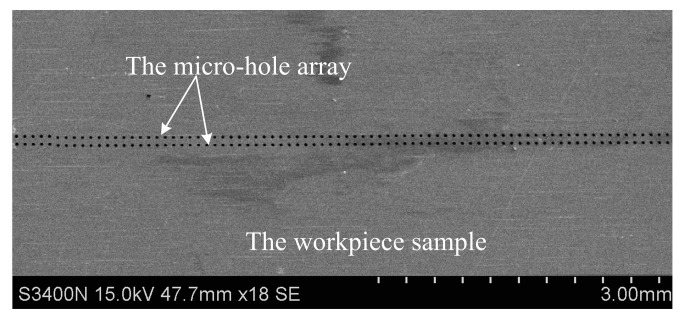
The SEM image of a 2 × 80 micro-hole array.

**Figure 16:**
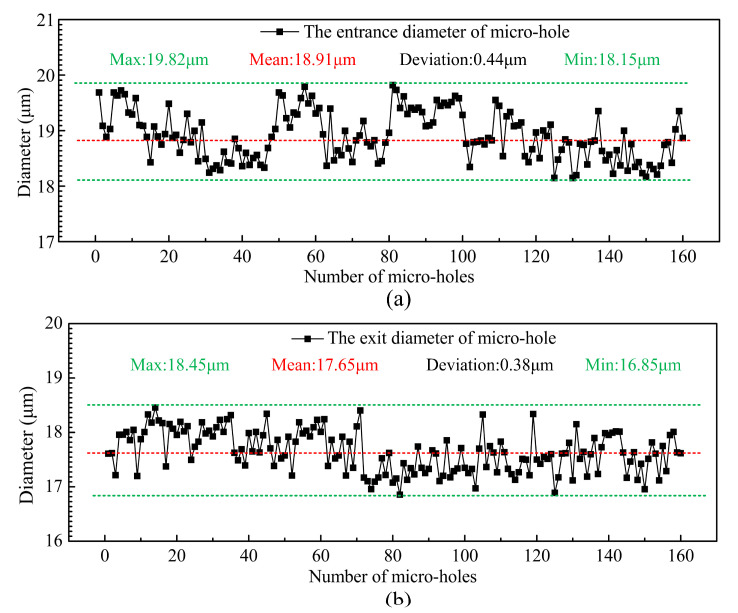
Measurements of the (**a**) entrance and (**b**) exit diameter of the micro-hole array.

Figure 15 shows the SEM image of a machined micro-hole array. Both the entrance and exit diameters of the micro holes were measured by a laser confocal microscopy (OLS4000, OLYMPUS, Japan) and the measured results were shown in Figure 16. The average entrance diameter of a total of 160 micro-holes was 18.91 μm with a 0.44 μm standard deviation (see Figure 16a) and the average exit diameter was 17.65 μm with a 0.38 μm standard deviation (see Figure 16b).

**Figure 17:**
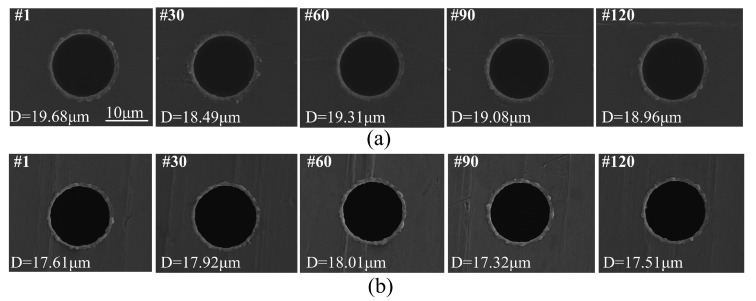
SEM images of #1, #30, #60, #90 and #120 micro-holes at (**a**) entrance and (**b**) exit side.

**Figure 18:**
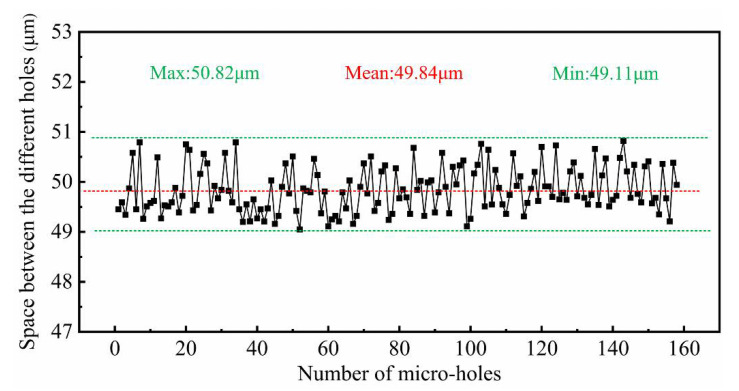
Space between the different holes of the micro-hole array.

Moreover, the taper rate (TR) was used to further evaluate the machined geometrical accuracy of micro-hole array, and the taper rate can be calculated by D’Urso et al. [27]:

(7) $$TR=\frac{\left(D_{entrance}-D_{exit}\right)}{H}$$

where $D_{entrance}$ is the entrance diameter of the micro-hole, $D_{exit}$ is the exit diameter of the micro-hole and H is the thickness of the workpiece. In this case, the range of taper rate (TR) was calculated to range from 0.0008 to 0.0575, which indicates good shape accuracy of micro-hole. The SEM images of the micro-hole (#1, #30, #60, #90 and #120) entrance and exit side were shown in Figure 17. The positioning accuracy of micro-hole array is ±1μm, as shown in Figure 18.

## 6. Conclusions

To machine ultra-small-hole array ($\Phi <20_{}\mu m$) with high consistency, accuracy and quality. The wire vibration of the WEDG system has been analyzed theoretically, and according to the established wire vibration model, an improved WEDG device was developed. Then, microelectrodes with a large aspect ratio and high consistency accuracy was fabricated. The effect of the applied voltage, current and pulse frequency on the quality of single micro-hole and microelectrode wear was also investigated. Within the limitations of the experimental conditions, the major conclusions of the study are summarized as follows:

1. The positioning sensor module has been used to reduce wire vibrations effectively. Micro-electrodes with ultra-small diameter (<15 μm), small diameter deviation (<0.4 μm) and large aspect ratio (142), which is the largest aspect ratio ever reported in the literature, were successfully fabricated.
2. A high applied voltage or a large machining current leads to an increase in micro-hole diameter and electrode wear as well as more recast materials on the surface of the micro-holes, while both the micro-hole diameter and electrode wear increased when pulse frequency was reduced. This may be caused by tool electrode resonance. It appears that one must avoid electrode resonance in order to benefit from the high accuracy and low electrode wear of the micro-EDM.
3. The results of the response–surface experiments showed that to fabricate micro-hole array with diameter 19 ± 1 μm using a single microelectrode, the applied voltage of 50 V, current of 80, pulse frequency of 250 kHz and pulse width of 3 μs were the optimal parameters.
4. By employing a touch measurement compensation strategy, the microelectrode tip can be assured to locate at the same starting point during the whole machining process. As a result, micro-hole array with high consistency and shape accuracy can be obtained.
5. Micro-hole array (2 × 80) with high consistency accuracy were successfully fabricated on a 50 μm thick stainless steel. The average entrance diameter of micro-holes array was 18.91 μm with a 0.44 μm standard deviation, and the average exit diameter of micro-holes array was 17.65 μm with a 0.38 μm standard deviation. The positioning accuracy of micro-hole array is ±1 μm.

## Figures and Tables

**Table 1:** Processing conditions for single micro-hole fabrication.

Parameters	Value				
Spindle speed (rpm)	600				
Voltage (V)	50	55	60	65	70
Current (index)	80	85	90	95	100
Frequency (kHz)	170	190	210	230	250
Pulse width (μs)	3				

**Table 2:** Micro-hole array fabrication parameters.

Parameters	Value
Applied voltage (V)	50
Current (index)	80
Frequency (kHz)	250
Pulse width (μs)	3
Safety distance (μm)	30
Feeding distance (μm)	93

## Data Availability

The data are not publicly available due to confidential.

## References

[1] Midolo L., Schliesser A., Fiore A. (2018). Nano-opto-electro-mechanical systems. Nat. Nanotechnol..

[2] Yi M.C., Feng J.B., Yin Z.F., Zou H.L. (2018). Fabricating method of SU-8 photoresist conical nozzle for inkjet printhead. Mater. Manuf. Process..

[3] Tong H., Li Y., Zhang L., Li B.Q. (2013). Mechanism design and process control of micro EDM for drilling spray holes of diesel injector nozzles. Precis. Eng..

[4] Klocke F., Klink A., Veselovac D., Aspinwall D.K., Soo S.L., Schmidt M. (2013). Turbomachinery component manufacture by application of electrochemical, electro-physical and photonic processes. CIRP Ann. Manuf. Technol..

[5] Zhang H.Q., Zhao S., Wang L.N., Li C., Hou S.X. (2019). Micro-drilling of AISI 1045 steel using a centering micro-drill. Int. J. Adv. Manuf. Technol..

[6] Chen T., Zhang G., Wang Y., Li X., Stoian R., Cheng G. (2020). Reconstructing of embedded high-aspect-ratio nano-voids generated by ultrafast laser bessel beams. Micromachines.

[7] Liu Y., Li M., Niu J., Lu S., Jiang Y. (2019). Fabrication of taper free micro-holes utilizing a combined rotating helical electrode and short voltage pulse by ECM. Micromachines.

[8] Wyszynski D., Bizon W., Miernik K. (2020). Electrodischarge Drilling of Microholes in c-BN. Micromachines.

[9] Yu Z.Y., Zhang Y., Li J., Luan J., Zhao F., Guo D. (2009). High aspect ratio micro-hole drilling aided with ultrasonic vibration and planetary movement of electrode by micro-EDM. CIRP Ann. Manuf. Technol..

[10] Routio M., Saynatjoki M. (1995). Tool wear and failure in the drilling of stainless steel. J. Mater. Process. Technol..

[11] Nasrollahi V., Penchev P., Batal A., Le H., Dimov S., Kim K. (2020). Laser drilling with a top-hat beam of micro-scale high aspect ratio holes in silicon nitride. J. Mater. Process. Technol..

[12] Heo J., Min H., Lee M. (2015). Laser Micromachining of Permalloy for Fine Metal Mask. Int. J. Precis. Eng. Manuf. Green. Technol..

[13] Fang X.L., Wang X.D., Wang W., Qu N.S., Li H.S. (2017). Electrochemical drilling of multiple small holes with optimized electrolyte dividing manifolds. J. Mater. Process. Technol..

[14] Manivannan R., Kumar M.P. (2017). Multi-attribute decision-making of cryogenically cooled micro-EDM drilling process parameters using TOPSIS method. Mater. Manuf. Process..

[15] Takahata K., Gianchandani Y.B. (2002). Batch mode micro-electro-discharge machining. J. Microelectromech. Syst..

[16] Chen S.T. (2007). A high-efficiency approach for fabricating mass micro holes by batch micro EDM. J. Micromech. Microeng..

[17] Hu Y.Y., Zhu D., Qu N.S., Zeng Y.B., Ming P.M. (2009). Fabrication of high-aspect-ratio electrode array by combining UV-LIGA with micro electro-discharge machining. Microsyst. Technol..

[18] Kim B.H., Park B.J., Chu C.N. (2006). Fabrication of multiple electrodes by reverse EDM and their application in micro ECM. Micromech. Microeng..

[19] Jahan M.P., Rahman M., Wong Y.S., Fuhua L. (2010). On-machine fabrication of high-aspect-ratio micro-electrodes and application in vibration-assisted micro-electrodischarge drilling of tungsten carbide. Proc. Inst. Mech. Eng. Part B J. Eng. Manuf..

[20] Gil R., Sanchez J.A., Ortega N., Plaza S., Izquierdo B., Pombo I. (2013). High-aspect ratio micro-pin manufacturing using inverse slab electrical discharge milling (ISEDM) process. Int. J. Adv. Manuf. Technol..

[21] Yin Q.F., Wang X.Q., Wang P., Qian Z.Q., Zhou L., Zhang Y.B. (2016). Fabrication of micro rod electrode by electrical discharge grinding using two block electrodes. J. Mater. Process. Technol..

[22] Masuzawa T., Fujino M., Kobayashi K., Suzuki T., Kinoshita N. (1985). Wire Electro-Discharge Grinding for Micro-Machining. CIRP Ann. Manuf. Technol..

[23] Li Y., Guo M., Zhou Z.Y., Hu M. (2002). Micro electro discharge machine with an inchworm type of micro feed mechanism. Precis. Eng..

[24] Zhang L., Tong H., Li Y. (2015). Precision machining of micro tool electrodes in micro EDM for drilling array micro holes. Precis. Eng..

[25] Li Z.K., Bai J.C., Cao Y., Wang Y.Q., Zhu G.Z. (2019). Fabrication of microelectrode with large aspect ratio and precision machining of micro-hole array by micro-EDM. J. Mater. Process. Technol..

[26] Alsahlani A., Mukherjee R. (2011). Vibration control of a string using a scabbard-like actuator. J. Sound. Vib..

[27] D’Urso G., Maccarini G., Quarto M., Ravasio C., Caldara M. (2016). Micro-electro discharge machining drilling of stainless steel with copper electrode: The influence of process parameters and electrode size. Adv. Mech. Eng..

